# Nanoparticle Boron Carrier for Boron Neutron Capture Therapy: Research Progress and Perspectives in China

**DOI:** 10.3390/nano16140845

**Published:** 2026-07-09

**Authors:** Haozhan Xie, Caiyun Fan, Fenglin Li

**Affiliations:** China Institute of Atomic Energy, Beijing 102413, China; 3180100082@zuaa.zju.edu.cn (H.X.); fancaiyun@ciae.ac.cn (C.F.)

**Keywords:** boron neutron capture therapy, nanoparticles, boron carriers, tumor targeting, drug delivery system

## Abstract

Boron Neutron Capture Therapy (BNCT), as a promising oncological modality, enables the specific therapy of tumor cells while minimizing damage to healthy tissues. It has emerged as a critical strategy for combating refractory malignancies such as glioma, breast cancer, lung cancer, and hepatocellular carcinoma. The development of efficient boron carriers is fundamental to realizing the clinical potential of BNCT. This review systematically traces the evolutionary trajectory of boron carriers, from first-generation soluble borates and second-generation agents to third-generation actively targeted formulations, with a particular focus on the current state of nanoparticle-based carriers. It provides a detailed analysis of the structural properties, boron-loading advantages, targeting modification strategies, and key research findings associated with various nanoplatforms, including liposomes, polymers, dendrimers, boron carbide nanoparticles, and gold nanoparticles. Furthermore, by examining specific tumor cell targets such as folate receptors and integrin receptors, the review elucidates the mechanisms by which nanocarriers achieve tumor boron enrichment through both the enhanced permeability and retention (EPR) effect and ligand-mediated active targeting. The review also critically assesses current challenges in the field, including targeting efficacy, boron loading capacity, in vivo retention, and biocompatibility. Finally, it summarizes emerging strategies—such as multi-target modification, combination immunotherapy, theranostics, and the induction of tumor cell pyroptosis—and provides a forward-looking perspective on future developments, aiming to inform the rational design of next-generation BNCT boron carriers with high targeting specificity, high boron payload, and low toxicity.

## 1. Introduction

Malignant tumors represent a major disease threatening the health of the Chinese population, with China currently experiencing low cancer cure and survival rates. Consequently, the innovation and advancement of oncological treatment technologies are urgently required. There is a critical need to develop safer therapeutic approaches for clinical oncology. High linear energy transfer (high-LET) radiotherapy has emerged as a research hotspot for treating refractory tumors due to its low dependence on cell cycle status and reduced incidence of radiation resistance. Within this category, Boron Neutron Capture Therapy (BNCT) demonstrates significant advantages by virtue of its unique binary treatment system.

Parallel to these developments, advances in nanotechnology, bioconjugation techniques, and tumor molecular biology have positioned nanoparticles as ideal carriers for BNCT boron delivery agents, owing to their distinctive physicochemical properties. Third-generation nanoparticle-based boron carriers are currently undergoing a paradigm shift from conventional “passive targeting” toward “active targeting” and “multifunctionalization.”

This review presents a systematic investigation of nanoparticle-based boron delivery systems for BNCT, comprehensively examining the research progress, targeting strategies, and therapeutic outcomes associated with various nanoboron carriers, while critically analyzing current challenges and exploring emerging research directions. Although several comprehensive reviews have provided an overarching landscape of BNCT boron carriers in general, the present review distinguishes itself by offering a systematic and critical analysis that spans both horizontal comparisons and vertical evolutionary trajectories across different nanoparticle-based boron delivery platforms. Furthermore, unlike previous international reviews, this work places particular emphasis on the research progress and clinical translation efforts within China, aiming to provide a regional perspective that complements the global understanding of the field, fosters international collaboration, and highlights the unique challenges and opportunities in the Chinese clinical context. In doing so, it also addresses the specific requirements of BNCT-related clinical parameters and discusses future development directions and potential solutions.

The overarching objective of this review is to establish a theoretical foundation for the development of high-efficiency nanoboron carriers suitable for clinical application, thereby facilitating the clinical translation of BNCT technology for the treatment of refractory malignancies. Ultimately, this work aspires to provide technical support for improving cancer cure rates and advancing the cancer prevention and control objectives outlined in the Healthy China initiative.

## 2. Status of Tumor Treatment

### 2.1. Current Status of Cancer Treatment in China

Due to the relatively high cancer mortality rate in China, incomplete statistics indicate that the five-year relative survival rate for malignant tumors from 2019 to 2021 was only approximately 43.7%, underscoring the urgent need for advancements in oncological research. This situation is particularly critical for brain tumors, lung cancer, liver cancer, and pancreatic cancer, which exhibit notably low cure and survival rates. The “Healthy China Action—Cancer Prevention and Control Action Implementation Plan (2023–2030)” sets a target of achieving a 46.6% overall five-year cancer survival rate in China by 2030 [1]. Consequently, the imperative to advance tumor treatment research is increasingly pressing.

Currently, radiotherapy has emerged as a focal point in oncological research, particularly high-LET radiotherapeutic approaches—including heavy ion therapy and neutron capture therapy—which have developed rapidly in recent years. These modalities exhibit low dependence on cell cycle status and a relatively low incidence of radiation resistance [2,3], offering therapeutic avenues for malignant tumors that are resistant to conventional treatments such as chemotherapy and X-ray radiotherapy.

From a clinical perspective, however, surgical intervention for malignant tumors—including brain tumors, lung cancer, liver cancer, and pancreatic cancer—frequently encounters numerous technical limitations and operational challenges [4]. Concurrently, both radiotherapy and chemotherapy are associated with significant adverse effects. In response to these limitations, BNCT has emerged as a promising oncological modality. This treatment approach enables effective tumor cell therapy while minimizing damage to normal tissues, aligning with the desired outcomes of cancer therapy. BNCT represents a significant advancement toward future tumor treatment—and potentially cure—offering substantial support for the extension of human life. Currently, BNCT has demonstrated notable clinical efficacy in the treatment of recurrent or unresectable locally advanced head and neck cancers, high-grade gliomas, and certain types of melanoma, although its application to other malignancies such as lung, liver, and breast cancers remains largely at the preclinical or early clinical investigational stage.

### 2.2. Tumor Treatment Targets

Therefore, to design drugs targeting tumor types with low cure rates, it is essential to leverage specific recognition sites on the tumor cell surface to achieve precise delivery and enhance drug accumulation directly at the tumor site. Table 1 lists several common surface targets on tumor cells with low cure rates, revealing that folate receptors and integrin family receptors are particularly prevalent. Leveraging these two targets enables the development of broad-spectrum antitumor drugs.

**Figure 1:**
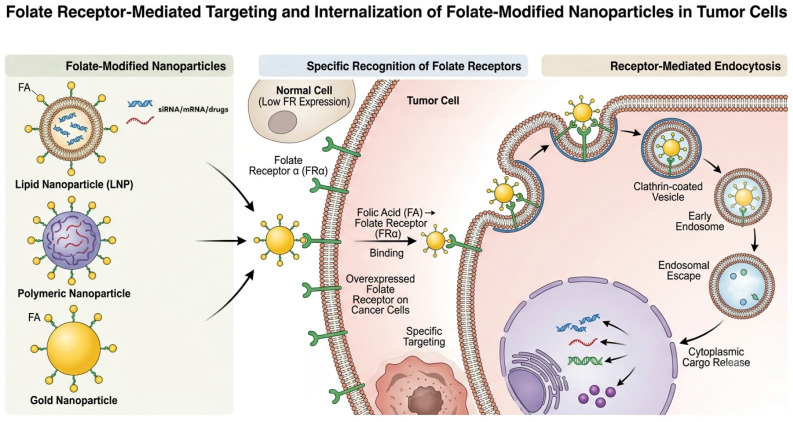
Boron carrier recognizes folate receptor.

Folate receptor is highly expressed on the surface of various tumor cells, making it an important molecule for tumor-targeted selection. The binding constant between folate receptor and its ligand folic acid is relatively high (Kd = 10^−10^ mol/L), and it exhibits favorable modifiability, enabling conjugation with various therapeutic agents. Constructing folic acid-modified nanodrug delivery systems can achieve folate receptor-mediated tumor-targeted drug administration [5]. Numerous studies have demonstrated the excellent targeting properties of folic acid-modified drugs. Ciofani et al. [6] synthesized boron nitride nanotubes (BNNTs) as boron carriers (Figure 1). They first coated the nanotubes with poly-L-lysine (PLL) to expose amino groups on the surface, then conjugated these with the carboxyl groups of folic acid and modified them with fluorescent quantum dots, ultimately obtaining functionalized BNNTs. This boron nitride nanotube drug delivery system was demonstrated to be selectively taken up by glioblastoma multiforme cells. Beyond BNNTs, Mandal et al. [7] utilized gold nanoparticles by alternately coating their surfaces with fluorescein isothiocyanate (FITC)-labeled ^10^B-polyallylamine hydrochloride (^10^B-FITC-PAH) and boronated sodium polystyrene sulfonate (^10^B-PSS), forming a multilayer polyelectrolyte shell. Finally, folic acid molecules were modified onto the particle surface through electrostatic interactions, yielding a functionalized gold nanoparticle boron delivery system that was shown to be internalized by various tumor cells.

**Figure 2:**
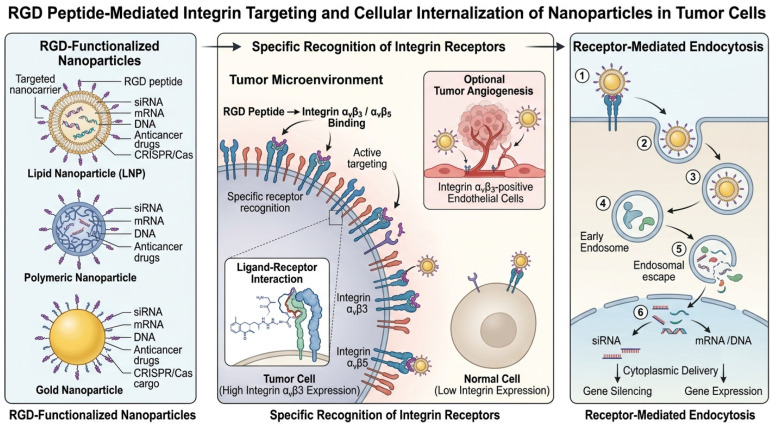
Boron carrier recognizes integrin receptor.

In addition, integrin receptor family members are also overexpressed on tumor cells (Figure 2). Integrin αvβ3 is highly expressed in most tumors and plays a crucial role in tumor angiogenesis and metastasis, making it one of the most attractive tumor targets. Peptides containing the Arg-Gly-Asp (RGD) sequence and their cyclic variants can specifically bind to αvβ3, establishing RGD as one of the most promising peptide sequences in tumor theranostics. c(RGDfV) was the first αvβ3 antagonist with high selectivity and activity, subsequently followed by the development of c(RGDfK) and c(RGDfC). In these structures, the lysine or cysteine residues can be modified with functional amino or sulfhydryl groups, and RGD-modified peptides have been demonstrated to achieve enhanced tumor targeting. Zhou et al. [8] prepared RGD-modified nanoliposomes containing quercetin. Animal experiments revealed that RGD-modified nanoliposomes could achieve localized accumulation in tumor sites of tumor-bearing mice, effectively inhibiting lung cancer cell growth. Amin et al. [9] employed dual-ligand modification of liposomes using RGD and TAT peptides to enhance liposomal targeting. This dual modification resulted in enhanced targeting levels both within and outside blood vessels, thereby improving the therapeutic efficacy of doxorubicin-loaded liposomes, although it also increased liposome clearance rates. Consequently, the primary tumor-specific recognition targets selected for this study are integrin receptors and folate receptors. Beyond surface targets, intracellular tumor targets have also emerged as research hotspots. Lei et al. [10] demonstrated through computational simulation that doxorubicin (DOX) possesses nuclear targeting capability and established that minor groove binding constitutes the predominant pre-binding step for DOX intercalation into DNA. During this binding process, local base pairs undergo opening and flipping; the space created by this flipping facilitates drug binding, and the resulting genetic misalignment triggers cell death. Efficient dual targeting of both extracellular and intracellular sites holds promise for achieving more precise cell killing.

### 2.3. Introduction of BNCT

**Figure 3:**
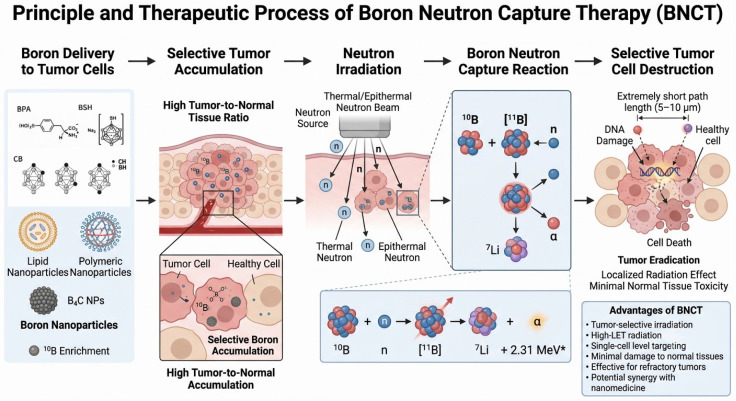
Schematic diagram of boron neutron capture therapy. (* Energies denoted in the figure are expressed in mega-electronvolts (MeV), where 1 MeV corresponds to 1.602 × 10^−13^ J, representing the kinetic energy released in the nuclear reaction.).

BNCT is a binary treatment system based on the interaction between ^10^B and thermal neutrons, first proposed by Locher in 1936 [11]. The stable isotope ^10^B possesses a neutron capture cross-section of 3835 barns (1 b = 10^−28^ m^2^), which is three orders of magnitude higher than that of other elements in the body. This property enables ^10^B to readily absorb low-energy thermal neutrons (<0.5 eV) and undergo nuclear reactions, producing α-particles and recoiling lithium atomic nuclei. The α-particles have an extremely short penetration distance of only 4–9 μm, which approximates the diameter of a single cell. By selectively delivering ^10^B to tumor cells and subsequently exposing them to neutron irradiation, specific killing of tumor cells can be achieved while sparing surrounding healthy tissue from damage [12] (Figure 3).

To ensure the therapeutic efficacy of BNCT, efficient uptake of boron carriers by tumor tissue must be achieved. Consequently, boron carriers should possess the following characteristics [13]: (1) Strong tumor targeting capability, with the ratio of ^10^B concentration in target tissue to that in non-target tissue (T/N ratio) exceeding at least 3–4:1; (2) High tumor uptake and prolonged retention time, with each tumor cell containing approximately 10^9 10^B atoms or achieving 20–50 μg ^10^B per gram of tissue at the time of neutron irradiation; and (3) Rapid blood clearance and low in vivo toxicity. Therefore, achieving high tumor cell uptake of boron carriers while minimizing toxicity represents the primary challenge currently facing BNCT boron carrier research and development.

## 3. Research of Boron Carriers

### 3.1. The First-Generation Boron Carrier

**Figure 4:**
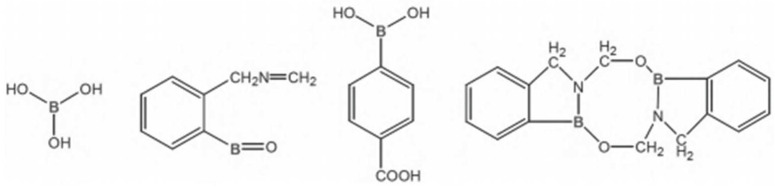
The first-generation boron carrier, boric acid, and its derivatives.

The evolution of boron carriers has now progressed to the third generation. The first-generation agents, comprising soluble borates (Figure 4), were utilized in early BNCT clinical trials but were subsequently abandoned owing to inadequate tumor targeting, insufficient intratumoral ^10^B accumulation, and fast blood clearance [14,15].

### 3.2. Second Generation Boron Carrier

**Figure 5:**
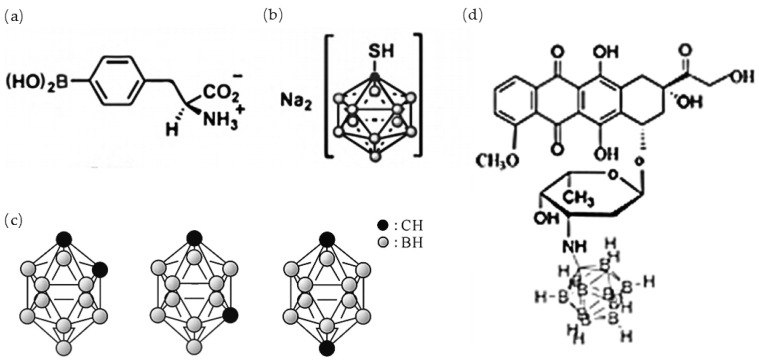
Part of the second-generation boron carriers: (**a**) L-BPA molecular structure diagram, (**b**) BSH molecular structure diagram, (**c**) CB molecular structure diagram, and (**d**) DOX and CB molecular structure diagram.

The second-generation boron carriers generally recognized as the most widely applied include 4-dihydroxyboryl-L-phenylalanine (L-BPA) and sodium mercaptoundecahydrododecaborate (BSH), along with boron-enriched agents such as carborane and doxorubicin-conjugated carborane (Figure 5).

In 1958, Snyder et al. [16] first synthesized L-BPA, which was initially employed for the treatment of melanoma [17]. Owing to its preferential uptake via amino acid transporters such as LAT1 that are abundantly expressed in neoplastic tissues, L-BPA can achieve selective accumulation in tumors. Subsequent studies by Yoshino et al. [18] and Li et al. [19] further confirmed its targeting efficacy and biodistribution profile. Currently, L-BPA has received official approval in Japan in 2020, representing the sole formally approved BNCT drug commercially available for the treatment of unresectable locally advanced or recurrent head and neck tumors, with extensive clinical data substantiating its efficacy as a boron delivery agent. However, its low boron content and susceptibility to intratumoral clearance continue to pose significant challenges that limit its broader clinical application.

In 1967, Soloway et al. [20] synthesized another boron carrier, BSH, which attracted considerable interest among BNCT researchers due to its high boron content. In 1968, Hatanaka et al. [21] pioneered the application of BSH in BNCT clinical trials, and subsequent studies demonstrated favorable therapeutic outcomes, gradually expanding its potential indications to include liver cancer, lung cancer, thyroid cancer, colon cancer, and even arthritis. Investigations by Li et al. [22] elucidated the metabolic pathways and toxicity profiles of BSH in vivo. Nevertheless, the structural and physicochemical properties of BSH result in limited tumor-penetrating capacity and relatively weak targeting specificity toward tumor cells, leading to inefficient cellular uptake. Moreover, high-dose administration increases the potential toxicity and adverse effects of the agent, which—along with similar limitations observed in other second-generation boron carriers—has precluded its regulatory approval for clinical use.

L-BPA and BSH have been employed as boron carriers in BNCT clinical research for over 70 years. Beyond objective factors such as limitations in neutron source production technology required for BNCT, ambiguous drug classification with attendant regulatory approval challenges, and substantial technical barriers arising from multidisciplinary integration, the second-generation boron carriers themselves exhibit inherent developmental limitations. These include suboptimal targeting specificity or insufficient boron content, as well as significant individual variability in tumor ^10^B uptake that renders L-BPA and BSH accumulation difficult to predict. Consequently, considerable variation exists in patient mean survival time (MST) following BNCT treatment, complicating efforts to establish standardized clinical therapeutic protocols.

Despite their limitations, L-BPA and BSH remain the only clinically available agents, providing critical experience and benchmarks for next-generation nanocarrier development.

### 3.3. Third Generation Boron Carrier

From the 1970s to the present, advances in drug synthesis technology, bioconjugation techniques, and tumor histopathology have ushered in a new era of boron carrier research characterized by high boron loading capacity and enhanced specificity, marking the advent of third-generation boron carriers. The third generation encompasses various boron carriers composed of targeting molecules conjugated with boron-containing compounds, including boron-containing amino acid analogs, boronated porphyrins, and nanoparticle-based boron carriers. The core design philosophy of third-generation boron carriers has shifted from “passive targeting” toward “active targeting” and “Multifunctionalization.”

Central to this evolution is precision active targeting, which manifests in two key aspects. First, it diversifies targeting strategies beyond traditional amino acid transporters, redirecting research focus toward tumor-specific targets such as folate receptors, prostate-specific membrane antigen, epidermal growth factor receptors, and integrins. Second, it incorporates high-affinity ligands to enhance tumor selectivity. A typical third-generation carrier comprises three functional modules: a targeting module, a boron cluster module, and a linker/modulator module. This modular architecture enables flexible optimization of individual components to balance targeting efficacy, boron payload, pharmacokinetics, and biodistribution. Beyond classical carboranes, researchers are exploring novel boron clusters—such as cobalt-containing carboranes—to increase both boron atom count per molecule and structural stability. Additionally, structures responsive to tumor microenvironmental conditions (e.g., low pH) are being introduced to achieve precisely controlled release, ensuring boron atoms remain localized at the site of action.

In summary, third-generation boron carrier research focuses on addressing fundamental challenges in boron delivery—selectivity, efficiency, and controllability—through precision molecular design. This represents a critical step in advancing BNCT from a promising technology toward a mature, reliable anticancer therapeutic modality. Although small molecule targeting agents constitute one core approach in third-generation design, numerous researchers contend that nanoparticle-based delivery platforms offer comprehensive advantages and greater promise as third-generation and even “next-generation” boron carrier vehicles.

The rationale for this perspective is multifaceted. First, nanoparticles address the “dosage” challenge by providing substantially higher boron loading capacity; a single nanoparticle (e.g., a liposome) can encapsulate numerous small-molecule boron drugs. Second, nanoparticles leverage the enhanced permeability and retention (EPR) effect, offering an additional layer of innate tumor targeting. Third, nanoparticles function as versatile multifunctional platforms amenable to flexible modification, enabling multifunctional targeting and precision delivery, ultimately facilitating theranostic applications. Fourth, nanoparticles (such as liposomes) provide protective and toxicity-attenuating effects; encapsulating potentially toxic boron compounds within nanoparticles reduces their exposure to normal tissues during circulation and mitigates side effects, while also protecting unstable boron agents from metabolic degradation before reaching the target site. Fifth, nanoparticle-based drugs (e.g., liposomal formulations) benefit from established technological expertise derived from successful clinical applications.

## 4. Nanoparticle Boron Carriers

Nanoparticles have garnered significant attention as pharmaceutical formulations due to their unique physicochemical and optical properties, as well as the nanoparticle-mediated EPR effect. The abnormal vascular structure and deficient lymphatic drainage at tumor sites result in enhanced permeability and retention phenomena for macromolecular drugs and nanoparticles within tumors.

Since its proposal in 1986, the EPR effect has become a crucial mechanism enabling macromolecular and nanomedicines lacking targeting ligands to achieve selective accumulation at tumor sites. It has also emerged as a fundamental prerequisite for constructing nanodrug delivery systems aimed at achieving tumor-targeted drug delivery. The combination of EPR effect-mediated passive targeting with diverse tumor-targeting ligands, along with inherent multifunctional expandability, has positioned nanoparticle-based systems as a research hotspot for novel boron carriers. The boronated porphyrin nanocomplex BPN developed by SHI et al. [23] exemplifies this approach, relying on the EPR effect to achieve tumor accumulation. Wang et al. [24] constructed a metal–organic framework structure based on zirconium (Zr) and tetracarboxyphenylporphyrin (TCPP), loading ^10^B-boric acid through host-guest reactions. This system utilized the EPR effect for targeted delivery to brain gliomas, achieving high boron accumulation in vivo (boron mass fraction > 60 ppm, where 1 ppm = 1 × 10^−6^), demonstrating considerable potential for BNCT applications. However, alternative research suggests that porphyrin-based drugs may not depend primarily on the EPR effect but rather utilize low-density lipoprotein (LDL) receptor-mediated delivery mechanisms [25], warranting further biological investigation.

Currently, nanoparticle-based boron carriers under investigation can be broadly categorized into several directions: liposomes, polymer-based nanoparticles, dendrimers, boron carbide nanoparticles, and gold nanoparticle-based boron carriers. These boron carrier types offer advantages including high flexibility, enabling conjugation with different targeting molecules according to specific tumor types, coupled with high boron content and substantial tumor boron accumulation. The following sections will provide systematic summaries of these nanoparticle-based boron carrier categories, addressing both their common characteristics and respective properties.

### 4.1. Liposome Boron Carrier

**Figure 6:**
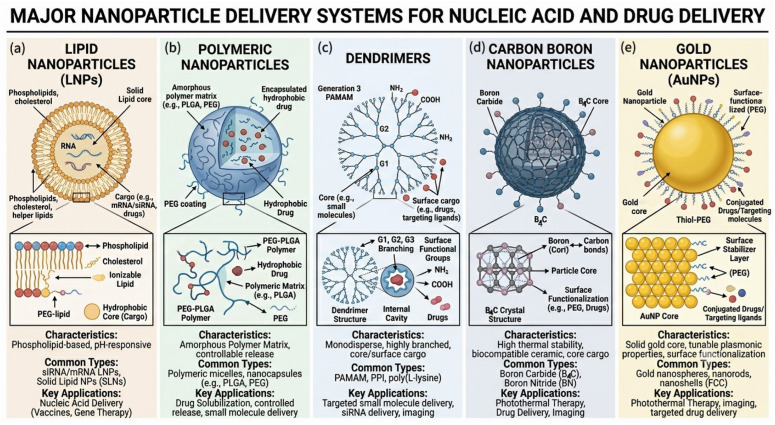
Major nanoparticle delivery systems: (**a**) Lipid nanoparticles (LNPs) and their brief introduction, (**b**) Polymeric nanoparticles and their brief introduction, (**c**) Dendrimers and their brief introduction, (**d**) Carbon boron nanoparticles and their brief introduction, and (**e**) Gold nanoparticles and their brief introduction.

Liposomes (Figure 6a) are nanoscale artificial membrane vesicles composed of phospholipid bilayers, with structures like biological membranes. Their ability to encapsulate both hydrophilic and hydrophobic substances for targeted delivery has established them as one of the most prominent nanoparticle-based boron carriers in current research [26].

Liposomes can also utilize the EPR effect to achieve tumor accumulation for boron-containing drug delivery, rendering them extensively studied. Representative investigations include Doi et al. [27] on transferrin receptor-mediated targeting, Kullberg et al. [28] on epidermal growth factor receptor-mediated targeting, Yanagie et al. [29] on multi-compartment liposomes prepared by coupling carcinoembryonic antigen (CEA) with BSH-encapsulating liposomes, and Chen et al. [30] on tumor cell nucleus-targeting liposomes loaded with the CRISPR-Cas9 system. These studies have all employed liposomes for ^10^B drug delivery, demonstrating that liposomes serve as relatively stable drug delivery vehicles as boron carriers.

Given their facile modifiability and biomembrane-mimetic properties, two principal strategies have emerged for improving liposomal boron drugs. The first involves coupling different specific targeting receptors to enhance active accumulation at tumor sites. The second focuses on improving encapsulated payloads to increase ^10^B content, achieving sufficient tumor boron concentrations (approximately 10^9 10^B atoms per tumor cell or 20–50 μg ^10^B/g tissue) while, ideally, maintaining controlled molecular size. Through recent years of research and development, liposomal drugs have evolved from first-generation drug encapsulation systems toward relatively mature therapeutic nanomedicines.

Liposomes represent the technological precursor to lipid nanoparticles (LNPs), with research dating back to the 1960s. Initially employed for encapsulating small-molecule drugs such as doxorubicin, they subsequently evolved to enable nucleic acid delivery. Subsequent phospholipid modifications introduced DOTAP (1,2-dioleoyl-3-trimethylammonium-propane), a permanently cationic lipid that maintains positive charge regardless of environmental pH, facilitating enhanced cell recognition and fusion, improving targeting, and strengthening interactions with negatively charged ions (e.g., nucleic acids). For instance, Chen et al. [30] utilized DOTAP-synthesized liposomes to encapsulate DOX-CB (doxorubicin-conjugated carborane) and plasmid DNA (pDNA) for combination immunotherapy. However, the relatively large particle size of the resulting liposomal nanomedicine presents limitations for brain tumor treatment. Alternative approaches include replacing pDNA with smaller mRNA and sgRNA combinations; Qiu et al. [31] employed LNP shells to achieve co-encapsulation and hepatocyte-specific delivery of Cas9 mRNA and sgRNA. Another alternative involves RNP (ribonucleoprotein) delivery, though this approach remains in the research phase due to stringent protein purification requirements.

While DOTAP facilitates cell fusion, it also introduces certain cytotoxicity; PEGylation offers partial mitigation but with limited efficacy. This limitation prompted the development of LNPs, which employ ionizable phospholipids that undergo protonation in the weakly acidic tumor microenvironment, thereby enhancing tumor targeting and cell fusion (facilitated by negatively charged cell surfaces). Numerous studies have applied such LNP shells. Baskararaj et al. [32] prepared folate receptor-targeted PEGylated liposomes delivering drugs to folate receptor-positive breast cancer cells. Their research demonstrated that folate-modified liposomes could target tumor cell mitochondria, significantly increasing reactive oxygen species (ROS) levels and impairing mitochondrial transmembrane potential, exhibiting substantial therapeutic potential. In recent developments, Liu et al. [33] proposed a unified framework decomposing LNPs’ complex, multifaceted characteristics into four independent yet interrelated functional domains, providing researchers with a systematic analytical and design language. This framework’s core value lies in transforming LNPs from a “black box” formulation into an understandable, predictable, and rationally designable modular platform.

However, LNPs still face challenges, with avoiding hepatic accumulation and improving tumor center penetration representing key directions for improvement research. Teng et al. [34] published a study in the Journal of the American Chemical Society entitled “Computationally Aided Design of Ionizable Cholesteryl Lipids for Lipid Nanoparticles to Modulate Hepatic mRNA Accumulation.” This research proposed a computer-aided ionizable lipid design strategy, developing and optimizing a class of sterol-derived ionizable lipids (iChol-lipids) with reduced ApoE (apolipoprotein E, mediating hepatocyte uptake) binding capacity. Compared to cholesterol, iChol-lipids exhibited significantly diminished ApoE affinity and could self-assemble with helper lipids and PEG lipids to form stable cholesterol-free three-component LNPs (Tc-LNPs). These achieved efficient mRNA delivery while substantially attenuating ApoE/LDLR-mediated hepatic accumulation, potentially supporting treatment of broader tumor types. Additionally, Mohammadzadeh et al. [35] investigated minoxidil liposomes for combination therapy. Addressing the challenge of nanochemotherapeutics exhibiting poor penetration and limited efficacy in dense tumors, they exploited minoxidil’s (MXD) vasodilatory properties, achieving targeted delivery via liposomes (Lip-MXD). Minoxidil, as a vasodilator, modulates the tumor microenvironment and inhibits tight junction proteins such as CLDN-1. Administering Lip-MXD prior to liposomal nanomedicine enabled enhanced penetration efficacy.

Determining when boron carriers achieve maximum accumulation in vivo is critically important for guiding clinical neutron irradiation timing. Qin et al. [36] constructed a bifunctional nanoliposome (BPA-F&DOTA-Gd@LIPO-ANG) capable of efficiently co-encapsulating therapeutic molecules (BPA) and imaging agents (gadolinium contrast agent), modified with targeting peptides to achieve integrated targeted delivery and real-time imaging. This enables real-time monitoring of tumor boron content to guide irradiation. Given that the radioisotope ^177^Lu is more readily available than gadolinium contrast agents in laboratory settings, radiolabeling approaches were employed to monitor boron distribution.

Consequently, liposomal boron carriers offer significant advantages compared to other third-generation boron carriers. As spherical nanodrug delivery systems composed of cholesterol and natural phospholipids forming bilayers, liposomes provide internal hydrophilic cavities and hydrophobic regions capable of loading substantial quantities of ^10^B compounds, including L-BPA, BSH, carborane, and DOX-CB. They exhibit prolonged intratumoral retention, which can be significantly enhanced through EPR effects and PEGylation. Preparation processes are straightforward, and liposome surfaces are readily modifiable. They demonstrate relatively favorable biocompatibility without inducing significant adverse reactions, undergo facile in vivo clearance without potential toxicity from long-term accumulation, and multiple antitumor liposomal formulations have already received marketing approval in China, demonstrating promising application prospects.

L-BPA and BSH have been employed in clinical research. Compared to L-BPA, BSH possesses higher boron content, prompting numerous studies on BSH-containing liposomes. However, BSH lacks targeting specificity and exhibits potential toxicity; additionally, the relatively high osmotic pressure within BSH liposome hydrophilic cavities can lead to premature BSH leakage. Conversely, BPA possesses inherent targeting properties and lower hydrophilic cavity osmotic pressure, precluding premature drug leakage, though its notably low boron content remains a significant limitation.

Current research is shown in Table 2, Table 3 and Table 4.

### 4.2. Polymer Boron Carrier

Polymer (Figure 6b) nanoparticles are widely employed in BNCT drug carrier research due to their straightforward preparation, high stability, well-controllable parameters, and facile functionalization characteristics. Among these, SUMITANI et al. [37] synthesized boron-containing polyethylene glycol-polylactic acid (PEG-PLA) block copolymers, which extended blood circulation time, demonstrated high tumor uptake rates, and featured simple preparation. PEG-PLA has currently achieved certain therapeutic effects in prostate cancer while exhibiting short drug half-life; this therapeutic approach also offers new treatment paradigms for malignant tumors beyond traditional drug delivery systems.

Boronated porphyrins can accumulate in tumors with prolonged retention, but clinical studies of relevant boronated porphyrins have revealed low tumor-to-blood (T/B) ratios and direct platelet toxicity, impeding their application in BNCT [38]. Under this premise, SHI et al. [23] utilized methoxy-polyethylene glycol-poly(lactic-co-glycolic acid) (mPEG-PLGA) micelles to encapsulate boronated porphyrin TBPP, preparing the boron-containing nanodrug BPN. BPN prevents direct contact between boronated porphyrins and blood cells, reducing off-target toxicity, and successfully delivers boron into the nucleus. Animal-level experimental results demonstrated that BPN almost completely inhibited melanoma growth in tumor-bearing mice, establishing it as a promising traceable boron carrier for future clinical melanoma applications [39].

The readily modifiable nature of dendrimers and polymers enables boron content enhancement through conjugation with multiple boron-containing molecules. For instance, Capala et al. [40] utilized the sulfhydryl groups of boronated PAMAM (BSD) to react with maleimide groups of EGF derivatives, producing a stable conjugate containing 960 boron atoms per molecule.

Compared with other nanoparticle-based boron carriers, polymeric nanoparticles offer exceptionally high boron-loading capacity and facile functionalization. However, their intrinsic structural features give rise to non-negligible biocompatibility concerns, which remain a major limitation for translational development.

### 4.3. Dendrimers as Boron Carriers

Dendrimers (DEs) (Figure 6c) as nanoparticle-based boron carriers have become ideal vehicles for BNCT-related therapeutic applications due to their facile modifiability and ability to significantly enhance drug loading capacity. Common dendrimers such as polyamidoamine (PAMAM) and polylysine feature branched structures and surface-modifiable groups that enable loading of high-density boron atoms (e.g., incorporating 20–50 boron groups per molecule). Additionally, their internal cavities can encapsulate boron compounds to enhance stability. Through conjugation with tumor-targeting ligands such as RGD peptides or folic acid, tumor specificity can be improved, achieving tumor-to-normal tissue (T/N) boron concentration ratios exceeding 3:1 [41,42].

pH-responsive dendrimers can also be employed to release boron drugs within the weakly acidic tumor microenvironment, minimizing leakage into normal tissues. Furthermore, given their facile modifiability, dendrimers can simultaneously carry both boron drugs and chemotherapeutic agents (such as paclitaxel) to enable combined BNCT and chemotherapy approaches.

As practical nanocarriers, block copolymers copolymerized with polyethylene glycol (PEG) can effectively prolong blood circulation time compared to BPA. Their hydrophobic cores can encapsulate poorly soluble drugs to enhance solubility, demonstrating strong drug-loading capacity. Consequently, they have been widely applied in drug imaging, targeted delivery, and cancer diagnosis and treatment [43].

Dendrimers similarly enable multi-site modification and ultrahigh boron loading, while offering a more precisely controllable molecular architecture than conventional polymers, thereby conferring superior functionalization efficiency. Nevertheless, their higher manufacturing costs and residual biotoxicity continue to impede clinical translation.

### 4.4. Boron Carbide Boron Carrier

Boron carbide particles (Figure 6d) feature simple and convenient synthesis methods, low toxicity, high boron content, excellent biochemical and thermal stability, and large specific surface areas with unique nanostructures that provide abundant adsorption sites and space for drugs, enabling functionalization and efficient drug loading. Consequently, they have been employed in BNCT research. Tsuji et al. [44] conjugated transferrin to boron carbide particles coated with poly-L-lysine and poly-γ-glutamic acid, forming spherical transferrin-conjugated boron carbide nanoparticles (Tf-SBCPs), which were subsequently labeled with fluorescent dyes. Following 2 h incubation with cervical cancer cells, Tf-SBCPs were extensively internalized into the cancer cells, whereas control SBCPs conjugated with albumin only accumulated on the cancer cell surface. When free transferrin was co-incubated with Tf-SBCPs and cervical cancer cells, Tf-SBCP internalization was competitively inhibited.

Boron nitride nanotubes (BNNTs) are structural analogs of carbon nanotubes. BNNT-based drug delivery can prevent drug degradation during delivery and prolong in vivo circulation time. Ciofani et al. [6] coated BNNTs with biocompatible poly-L-lysine (PLL) and subsequently surface-functionalized them with fluorescent dyes and folic acid, producing F-PLL-BNNT. Following 90 min incubation with glioblastoma cells, cellular uptake of F-PLL-BNNT was significantly higher compared to the PLL-BNNT control group.

Boron carbide nanoparticles possess the highest boron content among all nanocarrier categories, endowing them with exceptional capacity for delivering sufficient boron payloads to achieve effective post-irradiation tumor therapy. Their structural stability further favors therapeutic applications. However, the relatively high production costs present a substantial barrier to large-scale clinical manufacturing.

### 4.5. Gold Nanoparticle Boron Carrier

Gold nanoparticles (Figure 6e) offer advantages including facile surface modification and precisely controllable size, making them valuable for drug delivery applications. Pulagam et al. [45] functionalized AuNPs with boron-rich anions [3,3′-Co(1,2-C_2_B_9_H_11_)_2_]^−^, coated their surfaces with PEG, and performed ^124^I radiolabeling on both inner and outer layers for PET imaging. However, PET imaging in human fibrosarcoma-bearing mice revealed low tumor accumulation and poor tumor targeting of these AuNPs. Mandal et al. [7] coated AuNP surfaces with multilayered boronated polyelectrolytes comprising fluorescein isothiocyanate (FITC)-BPA-folic acid (FA), creating multifunctional AuNPs integrating ^10^B targeted delivery with in vivo imaging capabilities.

Human epidermal growth factor receptor 2 (Her2) is overexpressed on the cell membranes of various cancer types. Wu et al. [46] functionalized AuNPs with PEG, carborane, and azide compounds, subsequently conjugating them with anti-Her2 antibodies (61 IgG). Through click chemistry, they performed ^123^I radiolabeling of the AuNPs to obtain ^123^I-61-B-AuNPs. In gastric cancer-bearing mice, SPECT/CT imaging at 12 h post-administration demonstrated a tumor-to-muscle radioactivity ratio of approximately 12.02.

Gold nanoparticles, in contrast to polymeric, dendrimeric, and boron carbide platforms, exhibit superior theranostic capabilities, enabling the development of imaging-guided therapeutic agents as summarized in Table 3. Nonetheless, their limited batch-to-batch reproducibility arising from inherent instability represents a critical hurdle that precludes consistent clinical application.

To facilitate a more intuitive comparison, the core characteristics of the nanocarriers discussed herein are summarized in Table 5.

## 5. Emerging Strategies and Future Directions

Multi-targeting strategies involving multiple targeting groups and combination immunotherapy, as described above, may represent emerging directions for future research on nanoparticle-based BNCT drugs. By conjugating multiple targeting molecules, tumor targeting and retention can be enhanced. For instance, Takuya et al. [47] combined BNCT with immune checkpoint inhibitors (anti-PD-1 antibodies) for the treatment of melanoma. This boron neutron immunotherapy (B-NIT) approach not only suppressed tumor growth at the irradiated site but also elicited an abscopal effect on distal unirradiated tumors through the activation of CD8^+^ T cells and effector memory T cells, thereby successfully overcoming immunotherapy resistance. These findings demonstrate that B-NIT can induce potent antitumor immune responses even in immunotherapy-resistant models. In a separate study, Yang et al. [48] developed a platform based on lyophilized tumor cell-loaded ^10^B-doped carbon dots (^10^B-CDs), utilizing tumor cells simultaneously as antigen delivery vehicles and carriers for ^10^B-CDs. This represents the most recent investigation employing carbon dots—a subclass of quantum dots—as boron carriers for BNCT, and uniquely integrates boron delivery with tumor antigen presentation to achieve synergistic BNCT and immunotherapy. Chen et al. [49] from the Institute of High Energy Physics, Chinese Academy of Sciences, previously synthesized boron-containing carbon dots (BCDs) through hydrothermal conjugation of glucose and BPA. This structure simultaneously possesses the targeting characteristics of both glucose and BPA, enabling enhanced boron uptake in U87MG glioblastoma cells through the dual action of LAT1 and glucose transporters [50]. These findings suggest the feasibility of designing composite-targeting boron drugs, offering valuable references for targeting design in both small-molecule boron drugs and nanoboron carriers.

Additionally, combination therapy polymers can be developed by incorporating specific therapeutic moieties such as glycolysis inhibitors or ROS scavengers. Gao et al. [51] synthesized polymers containing reactive oxygen species (ROS) scavengers, with polymer monomers containing both BSH and ROS scavengers. Through polyion complex coupling, they obtained boron cluster-containing redox nanoparticles (BNPs). Following administration and neutron irradiation experiments, comparative analysis demonstrated that BNP tumor therapeutic effects far exceeded those of BPA, with no significant adverse reactions observed in mice after neutron irradiation. Qiu et al. [31] utilized LNP shells to achieve co-encapsulation and hepatocyte-specific delivery of Cas9 mRNA and sgRNA, combining pro-apoptotic factors with nanomedicine therapy. Li et al. [52] developed a ^10^B-enriched BNNS delivery system capable of loading doxorubicin (DOX) on its surface to form DOX@BNNSs. Notably, neutron irradiation was found to facilitate the release of DOX from the nanosheets, enabling a synergistic combination of BNCT and chemotherapy. In a 4T1 triple-negative breast cancer model, DOX@BNNSs combined with neutron irradiation achieved a tumor growth inhibition rate of 94.6%, significantly outperforming BNCT or chemotherapy alone. This study represents the first report of neutron-triggered drug release and demonstrates the potential of BNNS-based platforms for BNCT-chemotherapy combination therapy.

Recent studies also suggest that future BNCT may shift focus from simply increasing boron content toward utilizing BNCT as an immune-activating therapy. Wang et al. [53] discovered during boronate research that it can activate pyroptosis. Their experiments revealed that pyroptosis in less than 15% of cancer cells could achieve tumor immunosuppression and growth inhibition. In a review published in 2025, Mao et al. [54] systematically integrated the direct and indirect mechanisms of BNCT-induced cell killing and, for the first time, provided an in-depth exploration of the immunological potential of BNCT at both the molecular and cellular levels. The authors proposed that BNCT exhibits stronger immunogenicity than conventional radiotherapy and, when combined with immune checkpoint inhibitors, may achieve a synergistic therapeutic strategy characterized by “local activation and systemic response.” Therefore, future BNCT therapeutic emphasis may shift toward triggering tumor immune activation functions, where limited nuclear reactions at tumor sites mediate pyroptosis to achieve tumor treatment. This direction could potentially reshape requirements regarding BNCT drug boron content or the conventional T/N ratio threshold of greater than 3:1, achieving a paradigm of “treating partial tumor cells” to “activating immunity” to “systemic therapy”, establishing BNCT as an efficient trigger for immunotherapy.

Furthermore, studies have identified that tumor cells irradiated by BNCT can serve as “tumor vaccines” [55]. In tumor-bearing mice, after BNCT treatment eliminated the primary tumor, in situ re-challenge with tumor cells demonstrated inhibited tumor growth and prolonged animal survival, revealing additional possibilities for BNCT.

Beyond strategies focused on carrier design and targeting optimization, emerging computational approaches are also poised to accelerate BNCT research. For instance, recent advances in AI- and machine learning-enabled proteomics have demonstrated substantial potential in drug target identification, biomarker discovery, and drug repurposing [56]. Applying such proteomics-driven ML frameworks to BNCT could facilitate the identification of tumor-specific overexpression targets for boron carrier design, enable the discovery of predictive biomarkers for patient stratification, and support the repurposing of existing boron agents for new indications. This integration of computational proteomics with BNCT carrier development represents a promising yet underexplored direction for future research. Furthermore, beyond AI-assisted target identification, the same computational framework can be extended to the rational design of tumor-specific boron carriers, thereby establishing a complete design-to-delivery logic chain [57]. This integrated approach is expected to substantially shorten the upfront design phase of drug development and accelerate the transition to clinical research through expedited validation experiments.

## 6. Conclusions and Outlook

This review systematically examines the development trajectory of boron carriers for Boron Neutron Capture Therapy (BNCT), with particular emphasis on the current research status, structural characteristics, targeting strategies, and therapeutic outcomes of third-generation nanoparticle-based boron carriers. The following core conclusions have been derived:

As a precision oncological modality, the clinical efficacy of BNCT critically depends on the tumor targeting capability and boron enrichment efficiency of the boron delivery agents. First-generation soluble borates have been largely abandoned due to poor targeting specificity. Although the second-generation agents L-BPA and BSH have entered clinical investigation, they still fall short of meeting the clinical requirements of BNCT—L-BPA suffers from low boron loading capacity, whereas BSH exhibits weak tumor-penetrating ability and insufficient targeting specificity. These limitations may be closely associated with the impact of tumor heterogeneity on BNCT outcomes, as highlighted by Zhou et al. [58]. Consequently, the development of next-generation high-performance boron carriers has become an inevitable trend. For instance, Daniela et al. [59] reported a novel BNCT boron carrier based on an ortho-carborane-glucosamine derivative, which exhibits approximately threefold higher water solubility than the clinical gold-standard BPA and demonstrates significantly superior boron accumulation in U87-MG glioma cells with extremely low cytotoxicity. This study directly addresses the core clinical translational need for boron carriers that outperform BPA. In a separate study, Monica et al. [60] reported for the first time the synthesis and characterization of a novel ^10^B-enriched cobaltabis(dicarbollide) sodium salt, Na[o-^10^COSAN], and evaluated its biodistribution and therapeutic efficacy in a hamster oral cancer model. Although the absolute boron concentration in tumors was relatively low (approximately 11 ppm), Na[o-^10^COSAN]-based BNCT still induced significant antitumor effects, with the incidence of severe mucositis reduced by half and complete recovery achieved within 21 days.

Nanoparticle-based boron carriers have emerged as the core research direction for third-generation BNCT boron carriers, leveraging advantages including high boron loading capacity, inherent EPR effect-mediated targeting, multifunctional modification potential, biocompatibility, and toxicity attenuation through encapsulation. Recent surveys from 2025, such as the one conducted by Wan et al. [61], have also focused on nanoparticle-based agents for BNCT, expressing an optimistic outlook on their future development and proposing novel design strategies. Liposomes, polymers, dendrimers, boron carbide nanoparticles, and gold nanoparticles each possess distinctive characteristics. Liposomes, in particular, demonstrate the most promising application prospects due to their structural similarity to biological membranes, robust encapsulation capacity, facile surface modification, and established clinical technology accumulation. Dendrimers achieve high-density boron loading through branched structures, while boron carbide and gold nanoparticles exhibit unique advantages in surface functionalization and theranostic integration.

Folate receptors and integrin αvβ3 receptors overexpressed on tumor cell surfaces serve as important targets for active targeting of nanoboron carriers. Modification with targeting molecules such as folic acid ligands and RGD peptides significantly enhances tumor-specific uptake. Combining these modifications with the EPR effect enables “passive + active” dual targeting, effectively improving the ^10^B concentration ratio between tumor and normal tissues (T/N ratio). Furthermore, nanocarriers enable co-loading of boron drugs with chemotherapeutic agents and immunomodulators, providing a platform for combining BNCT with other therapeutic modalities.

Despite significant progress in nanoparticle-based boron carrier research, numerous common challenges persist: certain nanocarriers such as cationic liposomes exhibit cytotoxicity and off-target effects; LNPs are prone to hepatic accumulation and demonstrate limited tumor center penetration; some nanoboron carriers suffer from insufficient in vivo retention time and premature boron drug leakage; and real-time monitoring of in vivo boron enrichment requires further refinement, with standardized protocols for guiding clinical neutron irradiation timing yet to be established.

Emerging strategies including multi-target composite modification, tumor microenvironment-responsive release, theranostic integration, and combination immunotherapy offer effective approaches to address current research challenges. Examples include glucose-BPA conjugated boron-containing quantum dots achieving dual-targeting, pH-responsive dendrimers enabling controlled boron drug release, and bifunctional nanoliposomes facilitating targeted delivery with real-time boron content imaging—all significantly enhancing the application value of nanoboron carriers.

BNCT holds promise as a technology for precision tumor cure, with clinical translation fundamentally dependent on the development and optimization of nanoparticle-based boron carriers. Based on current research status and technological trends, future investigations will focus on the following directions:

Precision Targeting Design: Further exploration of tumor-specific targets and development of multi-target collaboration modified nanoboron carriers incorporating diverse targeting molecules including folic acid, RGD peptides, and monoclonal antibodies to achieve precise tumor cell recognition and efficient uptake. Concurrently, stimulus-responsive nanocarriers should be designed based on tumor microenvironment characteristics such as low pH, high glutathione levels, and specific enzyme expression, enabling controlled boron drug release at tumor sites while minimizing drug leakage into normal tissues, thereby enhancing targeting specificity and safety.

High-Performance Carrier Optimization: Structural improvement in existing nanocarriers to address current limitations, such as developing ionizable lipids with reduced ApoE binding capacity to resolve hepatic LNP accumulation; utilizing vasodilator modification or combination administration to improve tumor center penetration of nanoboron carriers; optimizing nanoparticle size, surface charge, and hydrophilicity/hydrophobicity balance to harmonize circulation time, tumor uptake efficiency, and blood clearance rate while reducing carrier cytotoxicity and immunogenicity.

Multifunctional Integration Development: Advancing theranostic integration of nanoboron carriers by combining ^10^B loading with imaging technologies including fluorescence imaging, MRI, and PET/SPECT to achieve real-time, precise monitoring of boron drug biodistribution, providing a standardized basis for clinical neutron irradiation timing and dosage determination. Additionally, leveraging nanocarrier multi-loading capacity to enable combined BNCT applications with chemotherapy, immunotherapy, and gene therapy—such as co-loading doxorubicin, cGAS-STING activators, or Cas9 mRNA—enhancing tumor killing efficacy through synergistic multi-modal approaches while overcoming tumor cell radiation resistance.

Innovative Boron Delivery System Development: Exploring novel conjugation methods between boron compounds and nanocarriers, developing boron clusters with high boron content and stability such as cobalt-containing carboranes to increase single-nanoparticle boron loading capacity; investigating emerging nanomaterials including metal–organic frameworks (MOFs) and quantum dots as boron carrier platforms, leveraging their unique structural characteristics for efficient boron loading and targeted delivery; simultaneously incorporating biomimetic technologies to develop erythrocyte membrane and cancer cell membrane-camouflaged nanocarriers, further enhancing biocompatibility and tumor targeting specificity.

Clinical Translation Advancement: Strengthening in vivo pharmacokinetic and toxicological investigations of nanoboron carriers to elucidate metabolic pathways, accumulation patterns, and potential adverse effects, establishing standardized biodistribution detection methodologies; conducting BNCT combination therapy experiments in small animal models to improve efficacy evaluation systems; promoting multidisciplinary integration combining nuclear physics, materials science, oncology, and clinical medicine research expertise to address challenges in neutron source production, drug regulatory approval, and clinical treatment standardization, accelerating clinical translation of nanoparticle-based boron carriers and advancing BNCT technology toward clinical application in refractory tumor treatment.

In summary, nanoparticle-based boron carriers present unprecedented opportunities for BNCT technology development. Through continuous innovation in targeting design, carrier optimization, and multifunctional integration, next-generation nanoboron carriers featuring high targeting specificity, high boron loading capacity, low toxicity, and theranostic capabilities will be developed. These advances will facilitate the establishment of BNCT as a conventional clinical modality for treating refractory tumors, providing robust technical support for improving five-year cancer patient survival rates and achieving the cancer prevention and control objectives of the Healthy China initiative.

## Figures and Tables

**Table 1:** Types of overexpressed receptors on the surface of four types of cancer cells.

Breast Cancer Cells	Lung Cancer Cells	Glioma Cells	Liver Cancer Cells
Human epidermal growth factor receptor	epidermal growth factor receptor	epidermal growth factor receptor variant	alpha fetoprotein receptor
Estrogen receptor	Anaplastic lymphoma kinase	Platelet-derived growth factor receptor	Platelet-derived growth factor receptor
Folate receptor	Folate receptor	Folate receptor	Folate receptor
Integrin receptor family	Integrin receptor family	Integrin receptor family	Integrin receptor family

**Table 2:** Current research status of different liposomal drugs.

Research Title	Core Carrier	Drug Loaded	Key Findings
cGAS-STING activator liposomes (Wang et al., [24])	Ultrasound-responsive liposomes	Low-dose doxorubicin (DOX)	Primarily induces immunomodulation
Double Modified Liposomes (Amin et al., [9])	Liposomes	Doxorubicin (DOX)	Double Targeted Enhancement but Increased Blood Clearance
RGD (integrin receptor) modified cationic liposomes (Chen et al., [30])	PEGylated cationic liposome	DOX-CB complex	RGD exhibits good targeting, but cationic liposomes bring off-target effects
pH sensitive dual targeted liposomes	pH-responsive phospholipid liposomes	Multiple chemotherapy drugs	pH-responsive targeting is good, but the preparation process is difficult
Folic acid targeted PEG liposomes (Baskararaj et al., [32])	PEGylated liposomes	Seaweed bioactive compounds	Folic acid has good targeting ability and can target mitochondria
CD24 antibody targeted liposomes	liposome	cisplatin	Antibodies have good specificity, but their stability is insufficient, and production costs are high

**Table 3:** Study on radioactive labeling of AuNPs and liposomal drugs.

Research Title	Core Carrier
Pulagam et al. conducted ^124^I labeling on AuNPs (kind of gold nanoparticles) for PET imaging	gold nanoparticles
Wu et al. labeled AuNPs with ^123^I for SPECT/CT imaging	gold nanoparticles
Qin et al. constructed bifunctional nanoliposomes and used gadolinium (Gd) for MRI imaging to monitor tumor boron content in real time	Connecting liposomes through DOTA (kind of linker)

**Table 4:** Horizontal comparison of different liposomal drugs.

Comparative Dimension	Liposome	Cationic Liposome	LNPs (Lipid Nanoparticles)
Core lipid composition	Mainly composed of traditional phospholipids (such as phosphatidylcholine)	Permanent cationic lipids such as DOTAP	Ionizing cationic lipids (can be further improved to reduce lipids targeting liver cells)
pH response	/	/	Protonation in acidic environment
Application scenarios	Early use of small molecule chemotherapy drugs (such as doxorubicin liposomes) with limited application scenarios	Partial liposome therapy drugs	Due to its outstanding nucleic acid delivery capability, it has been widely used in mRNA vaccines and gene therapy, and is currently the mainstream platform for nucleic acid drug delivery
Biocompatibility and cytotoxicity	Low toxicity: neutral or slightly negatively charged at physiological pH, with weak interaction with the cell membrane	Cationic liposomes are prone to trigger immune reactions and toxicity, and although PEGylation can improve them, the effect is limited	CILs replace traditional cationic lipids, significantly reducing toxicity and inflammatory response
Stability and internal escape	Poor stability: prone to exchange and fusion with blood components (such as lipoproteins), low efficiency of endosome escape	High stability, insufficient internal escape efficiency	High stability, PEGylated lipid shell provides “invisibility” ability, prolongs blood circulation time, and high efficiency of endosome escape

**Table 5:** Comparative analysis of different nanocarriers.

Platform	Boron Loading Capacity	Targeting Strategy	Key Advantages	Critical Bottlenecks	Clinical Translation Stage
Liposome/LNPs	Moderate–High, can encapsulate both hydrophilic and hydrophobic drugs	EPR effect + ligand modification (e.g., folate, RGD, Tf, antibodies); ionizable lipids for pH-responsive targeting	Biomembrane-mimetic, good biocompatibility, versatile payload encapsulation (small molecules, nucleic acids), established manufacturing experience, surface easily functionalized	Premature drug leakage, limited tumor penetration (especially in dense tumors), hepatic accumulation of LNPs, cationic lipids may cause cytotoxicity	Clinically approved (e.g., DOX-liposomes); BNCT liposomes in preclinical/early clinical
Polymer (e.g., PEG-PLA)	Moderate (boron clusters or porphyrins loaded in hydrophobic core; encapsulation efficiency varies)	EPR effect + ligand conjugation (e.g., folate, RGD); prolonged circulation via PEGylation	High stability, tunable degradation kinetics, good drug-loading capacity, well-established synthesis, reduces off-target toxicity (e.g., porphyrin toxicity shielded)	Drug half-life may be short; batch-to-batch reproducibility challenges; limited in vivo tracking capability	Preclinical (some formulations in early-phase clinical trials for other indications, but not yet for BNCT)
Dendrimers (e.g., PAMAM)	High (20–50 boron clusters per molecule via surface conjugation; up to 960 B atoms/dendrimer reported)	Ligand conjugation (folate, RGD, EGF) + pH-responsive release; passive accumulation via EPR	Highly branched structure enabling high-density boron loading; precise molecular architecture; internal cavities for additional encapsulation; multifunctionalizable	Complexity in synthesis and purification; potential immunogenicity; scale-up challenges; unclear metabolic fate and long-term toxicity	Preclinical
Boron Carbide (e.g., BNNTs)	Very High (BNNTs possess high B/C ratio)	EPR effect + surface functionalization with targeting ligands (e.g., Tf, folic acid, PLL coating)	Extremely high boron content; excellent thermal and chemical stability; large surface area for functionalization and drug loading	Limited biodegradability; potential long-term retention; insufficient in vivo tracking data; unclear biodistribution and clearance pathway	Preclinical (very early stage)
Gold nanoparticle (e.g., AuNPs)	Moderate (boron-rich anions or carboranes conjugated on surface; layer-by-layer assembly can enhance loading)	EPR effect + active targeting (e.g., folate, Her2 antibody, RGD); surface plasmon resonance enabling theranostic integration	Excellent surface modifiability; precise size control; intrinsic imaging potential (CT, PET/SPECT via radiolabeling); enables theranostic applications	Low tumor accumulation reported in some studies; relatively low boron payload per particle; high cost; unclear long-term toxicity and clearance	Preclinical (with some radiolabeled formulations undergoing imaging studies)

## Data Availability

No new data were created or analyzed in this study.
